# A Pipeline Program to Address the South African Crisis in Human Resources for Health

**DOI:** 10.29024/aogh.12

**Published:** 2018-04-30

**Authors:** Kalay Moodley, Tabia Henry Akintobi, Therese Fish, Daniel S. Blumenthal

**Affiliations:** 1 Stellenbosch University, Faculty of Medicine and Health Science, ZA; 2 Morehouse School of Medicine, GE

## Abstract

**Background:**

The WHO Africa Region faces a shortage of health workers due to inadequate production of health workers and emigration of physicians and nurses to wealthier countries. South Africa and the United States share a history of discriminatory policies and practices resulting in groups that are under-represented amongst health professionals. One US response is the Area Health Education Centers Program (AHEC), a pipeline program to recruit members of under-represented groups into the health professions.

**Objectives:**

(1) Compare and contrast the United States’ AHEC model with that developed in South Africa by Stellenbosch University Faculty of Medicine and Health Sciences SA AHEC in partnership with Morehouse School of Medicine in the United States. (2) Describe a formative evaluation of the Stellenbosch AHEC Program.

**Methods:**

Four hundred students (grades 7–12) and 150 teachers participated in SA AHEC with the goal of preparing the students to better compete for university admission. Students received after-school tutoring, holiday schools, and counselling on study skills, health careers, and university entry. Educators received continuing professional education, classroom observation, and feedback. The program was evaluated through a series of interviews and focus groups involving AHEC staff, educators, and parents and caregivers.

**Results:**

Program strengths included educator training, collaboration, and increased student maturity, motivation, and academic success. Challenges included limited time with students, the location of some sites, and the educators’ need for more engagement with AHEC staff and schools. Quarterly workshops were conducted to address challenges. Over 50% of program alumni are currently enrolled in institutions of higher education. Students will be tracked to determine whether they are able to complete their health professions studies and return to the communities where they grew up, or to similar communities.

**Conclusions:**

With appropriate adaptation and attention to context, it might be possible to implement similar programmes in other African countries. The comparison of the United States and South African models suggested that more parent and teacher participation in an advisory capacity might help to avoid some challenges.

## Background

The WHO Africa Region faces a severe and chronic shortage of health workers, with over 800,000 physicians and nurses alone required to meet estimated minimal needs [1]. This shortage is due both to inadequate production of health workers and emigration of physicians and nurses to wealthier countries (the “brain drain”).

The situation is less dire in South Africa than in most other sub-Saharan African countries, with 0.776 physicians per 1000 population and 5.114 nurses per 1000. This compares favourably with (for instance) 0.40 per 1000 and 3.35 per 1000, respectively, in neighbouring Botswana and (as an example of a less-developed country) 0.107 per 1000 and 0.529 per 1000, respectively, in the Democratic Republic of Congo. However, South Africa lags far behind developed countries such as the United States, which has 2.3 physicians per 1000 population [2] and 9.4 nurses per 1000. Nonetheless, health policy experts in the United States consider that it has a shortage of physicians and nurses [3].

The situation is complicated in both South Africa and the United States by a history of discriminatory policies and practices that have resulted in population groups that are under-represented amongst health professionals: African Americans and Hispanics in the United States and blacks and coloureds in South Africa. These are population groups that, in both countries, suffer from disparities in health and health care; these are disparities that could be addressed, at least in part, by increasing the number of physicians and other health professionals who are drawn from the affected groups, because these health workers are the most likely to serve the affected groups [4,5]. The maldistribution of physicians by race and ethnicity is shown in Tables 1 and 2. Similarly, in both countries (as in virtually every country in the world), there is a relative shortage of physicians and other health care personnel in rural and low-income communities. Although 43.6% of South Africa’s population resides in rural areas, they are served by only 12% of the country’s doctors and 19% of its nurses [6].

**Table 1:** Distribution of Physicians and Population by Population Group, United States.

Population Group	Physicians* N = 471,408	Population** N = 308.7 million

Total	100.0%	100.0%
White	75%	63.7%
Black or African American	6.3%	12.2%
American Indian and Alaska Native	0.5%	0.7%
Asian	12.8%	4.7%
Hispanic or Latino	5.5%	16.3%

*Source: Castillo-Page L. Diversity in the Physician Workforce Facts & Figures 2010. Washington DC: AAMC; 2010. **Source: US Census Bureau: National Population Estimates; Decennial Census.

**Table 2:** Distribution of Physicians and Population by Population Group, South Africa.

Population Group	Physicians* (2008) N = 34,324	Population (2011 census) N = 51.58 million

White	44.8%	9.1%
African (Black)	15.0%	76.4%
Coloured	1.4%	8.9%
Indian (Asian)	12.4%	2.4%
Race not specified or other	26.4%	0.5%

*Source: The Shortage of Medical Doctors in South Africa. Scarce and Critical Skills Research Project. Research commissioned by the Department of Labour, South Africa; March 2008.

An examination of the leading causes of death in each population group helps to demonstrate the impact of the human resource crisis (although it must be recognized that medical care is only one of many factors affecting mortality). Table 3 lists the top 10 causes of death for each population group in a recent year. The leading causes of death in whites are similar to those in developed countries: heart disease, stroke, cancer. The same is true for the Indian/Asian population, with the addition of diabetes mellitus in first place. On the other hand, the leading causes of death in the black population are those of developing countries: tuberculosis, pneumonia/influenza, and diarrhea; five of the top 10 are infectious disease. The distribution of causes of death in the coloured population is a blend of the other two, with tuberculosis in first place, followed by diabetes mellitus and stroke.

**Table 3:** Top Ten Causes of Death, South Africa, 2010.

Cause	Black	White	Indian/Asian	Coloured	Unknown

TB	1		10	1	1
Influenza & Pneumonia	2	7	9		2
Intestinal Infectious Disease	3				3
Other Heart Disease	4	2	3	9	4
CVA	5	3	4	3	5
HIV	6			7	6
Diabetes mellitus	7	6	1	2	7
Hypertensive Disease	8	9	7	10	8
Other Viral Disease	9				9
Immunological Disorder	10				10
Ischemic Heart Disease		1	2	5	
Digestive System Cancer		4	5	8	
COPD		5	6	4	
Lung Cancer		8	8	6	
Renal Disease		10			

Source: Statistics South Africa.

This reflects the distribution of the proportionate representation of physicians in the population groups (Table 2). White and Indian physicians are overrepresented; for instance, 44.8% of South African physicians are white, although whites comprise only 9.1% of the South African population. By contrast, black and coloured physicians are underrepresented; for instance, only 15% of South African physicians are black, although blacks comprise 76.4% of the South African population.

One approach that has been used in the United States with some success to address the racial/ethnic and geographic shortages of human resources for health is a pipeline program known as an Area Health Education Centers (AHEC) Program [7]. In this paper, we describe the AHEC Program as it has developed in the United States, describe a pilot AHEC Program in South Africa, discuss the differences in the two models, and offer a formative evaluation of the South African program. The South African program represents the first attempt at establishing an AHEC Program outside of the United States.

### AHEC in the United States

The US Area Health Education Centers Program was first funded by Congress in 1971 to help address the shortage of physicians in rural areas. A Carnegie Commission report recommended the creation of AHECs at regional hospitals to offer some of the functions of academic medical centres, such as teaching and continuing education, suggesting that this would encourage physicians to remain in small towns nearby. As funded by the federal government, AHEC evolved into a pipeline program designed to attract rural and minority schoolchildren into the health professions, provide a portion of their professional training in underserved communities (communities with inadequate health services), and support them after graduation through continuing education and other activities once they had established practices in communities similar to those from which they had come.

**Figure 1:**
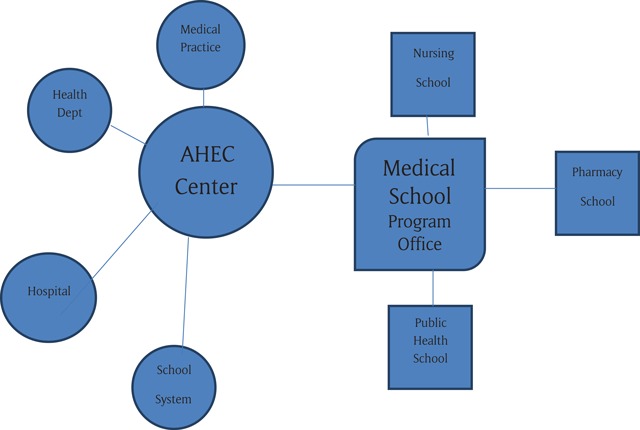
United States AHEC Model.

In the current model, federal funds flow through medical schools to affiliated AHEC centres, which are organizations either housed in regional hospitals (as in the original Carnegie proposal) or independently incorporated as not-for-profit corporations. The AHEC centres have advisory boards (those that are hospital based) or boards of directors (those that are independent corporations) on which are represented nearby hospitals, medical practices, public health departments, community health centres, and other health care organizations, as well as consumers. The centres conduct presentations on health careers to schoolchildren, arrange preceptorships for medical and other health professions students, and sponsor continuing education programs. Support and general oversight of the centres is provided by the program office at the medical school. An AHEC Program may have as few as one or as many as nine affiliated centres. Interprofessional training is encouraged and may be achieved through partnerships between the medical school and the other professional schools in the academic health centre (Figure 1).

Currently, federal funding is intended to be focused on start-up activities and initial support. After a specified number of years, each program is expected to obtain the majority of its funding elsewhere. Typically, this is from state government, although support may be obtained from private sources. If the program is successful in obtaining non-federal funding, it remains eligible for limited federal dollars.

### AHEC in South Africa

In 2010, the Stellenbosch University Faculty of Medicine and Health Science (FMHS) received a grant from the United States Health Resources and Services Administration (HRSA) to support its participation in the Medical Education Partnership Initiative (MEPI). MEPI was a component of the President’s Emergency Program for AIDS Relief (PEPFAR) that supported medical schools in 13 sub-Saharan African countries, with academic partners in the United States to “advance PEPFAR’s goal of increasing the number of new health care workers by 140,000; strengthen in-country medical education systems; and build clinical and research capacity in Africa as part of a retention strategy for faculty of medical schools and clinical professors” [8]. In 2011, the MEPI grant at Stellenbosch was augmented with another HRSA grant to establish an AHEC Program. Morehouse School of Medicine was designated as a partner on the grant with chief responsibility for evaluation; Morehouse had had an AHEC Program since 1984.

The main Stellenbosch University campus is located in the town of Stellenbosch, 35 kilometres from Cape Town, while FMHS is housed in Cape Town in the 1,384-bed Tygerberg Hospital and adjacent buildings. The faculty offers degree-granting programs in medicine, nutrition, nursing, occupational therapy, physiotherapy, and speech-language and hearing therapy. Entry to these programs, including medicine, normally follows graduation from secondary school (i.e., completion of grade 12).

In recent years, Stellenbosch has become a bilingual institution with some lectures given in Afrikaans and others in English, and in the post-apartheid era, it has become racially integrated. FMHS has taken steps to become more “socially accountable” [9]. One element of this effort has been the development of the Ukwanda Rural Clinical School (RCS), which includes a mini-campus located in the town of Worcester, 113 kilometres from Cape Town. It is near Avian Park, a large, low-income community where some vineyard workers live, in addition to many families with no source of employment. The mini-campus has an educational building and comfortable housing for 40 students. Clinical teaching sites are accessed through partnerships with the nearby regional hospital, a tuberculosis hospital, 7 more distant regional hospitals, and 70 clinics, both fixed and mobile. The RCS provides interdisciplinary educational opportunities for students in all of the fields that are part of FMHS; all final-year medical students rotate briefly through the school, and year-long rotations are offered on an elective basis.

An additional element of the socially accountable strategy at Stellenbosch is the development of a pipeline to encourage and facilitate the enrolment of underrepresented population groups in its educational programs. The MEPI grant provided financial support for the pipeline but did not provide financial support for the first component, the portion that encourages and helps prepare schoolchildren from underserved communities and population groups to compete for places in professional school. The AHEC grant enabled the development and implementation of this component.

**Figure 2:**
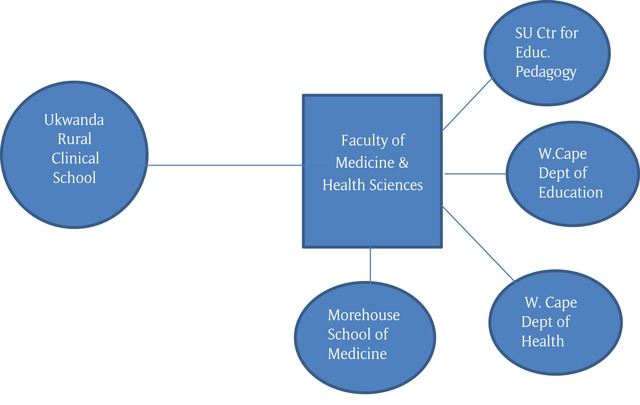
South Africa AHEC Model.

The AHEC Program was centred at the RCS, but unlike the US AHEC model, the RCS was not independent of the university. The program’s advisory board included representatives of the government’s Departments of Health and of Education, the Stellenbosch University Faculty of Medicine and Health Sciences, its Center for Educational Pedagogy (SUNCEP), and a representative of the Morehouse School of Medicine (Figure 2).

The AHEC intervention served schools in three rural districts of the Western Cape Province: Caledon, Malmesbury, and Worcester. It represented a collaboration between FMHS and the Stellenbosch University Center for Educational Pedagogy (SUNCEP). Over four years, 400 (mostly coloured, with few blacks or Indians) with above-average grades and 150 teachers enrolled in an intervention that offered the following:

- Grades (school years) 7–9: Bi-weekly after-school sessions in which teachers served as tutors to provide additional instruction in science and mathematics. Thirty students per district participated each year.
- Grades (school years) 10–12: Thirty students per district attended “holiday school” – additional instruction in science and mathematics in one-week sessions offered three times per year during vacation periods.
- Life skills: All participating students received career counselling, visits to Stellenbosch University, instruction on study skills, information about scholarships and the university application process, and other supports to help them prepare for university entry.
- Teacher continuing professional education: Training sessions in mathematics and science were provided by SUNCEP faculty to teachers participating in the intervention. This was followed by classroom observation and feedback.

### Comparison of the Two Models

Table 4 compares and contrasts, the two models. As noted above, the US model calls for an independent AHEC centre with its own governance structure that is remote from the medical school, whereas in the South African model, the remote site is a mini-campus of the medical school. Most US AHEC programmes attempt to “recruit” black and Hispanic children from rural or inner-city communities into health professions through presentations at their schools and summer and weekend activities. These initiatives do not usually affect the schools’ teaching programmes. In South Africa, by contrast, the focus is on the education of the students; this is impacted through teacher training and supplemental educational opportunities for the students.

**Table 4:** Comparison of US and South Africa AHEC Models.

	US Model	South Africa Model

Educational Site	Remote AHEC centre that is independent of medical school	Remote medical school mini-campus
Governance	AHEC centre board of directors, or host regional hospital with advisory committee	Faculty of medicine and science with advisory committee
Pipeline Programme	Promote health careers amongst underrepresented* primary and secondary school students	Strengthen educational experience for underrepresented* secondary school students
Programme Conducted By	AHEC centre staff	University faculty of education; secondary school teachers
Advisory Board or Board of Directors	Representatives of medical school, other academic institutions, health departments, medical practices, hospital, consumers	Representatives of faculties of medicine and science and education; national departments of health and education; Morehouse School of Medicine (US partner)
Stakeholders	Underserved communities, parents, students, health professions schools	Underserved communities, parents, students, health professions school

*Underrepresented students: students from population groups whose percent representation amongst physicians is significantly less than their representation in the population as a whole. US: black and Hispanic. South Africa: black and coloured. Both countries: rural.

Both the United States and South African AHEC programmes have advisory boards or similar structures, but the US committees are more diverse and include institutions and agencies outside of the medical school, as well as consumers. Both models call for an interdisciplinary approach, and the stakeholders are essentially the same.

## Methods: Evaluation Approach

Three years after program initiation, an evaluation was conducted by the Morehouse School of Medicine Evaluation and Institutional Assessment (EIA) Unit. This is an independent, institutionally designated entity designed to evaluate the degree to which programs and partnerships have achieved strategic aims, goals and objectives. The EIA Unit’s approach has been applied to community-based, regional and national evaluations [10,11,12,13,14]. The EIA Unit worked with the AHEC project director to initiate a process towards execution of the external evaluation plan in 2014. A second evaluation, focused on the students, was conducted in 2016 and will be the subject of a subsequent report.

The goal of this process assessment was to garner AHEC model and implementation perceptions, experiences, and recommendations from three stakeholder groups, including (1) SUNCEP staff, (2) educators, and (3) parents and caregivers. Key informant interviews were conducted amongst SUNCEP staff who were central to implementation of the AHEC program due to their history and leadership in preparing teachers and learners for careers in science and mathematics. Tutors and curriculum coordinators were engaged in key informant interviews through solicitation and recruitment by SUNCEP staff. Parents or caregivers of learners were essential to identifying community perceptions and attitudes regarding AHEC, as well as approaches central to improvements. The EIA Unit visited Stellenbosch and AHEC sites in March 2014.

Analysis of key informant interviews and focus groups were preceded by transcription of interviews. Interviews were manually coded by at least two EIA Unit members. Once responses were independently coded, team members met to consolidate findings towards thematic analysis [15]. Instances of theme discrepancy were discussed until a consensus was reached. To guide analysis of the results, key themes were determined following coder consensus. Discussions were transcribed to initiate manual data analysis supported by NVIVO 9.0 qualitative data analysis software. Both stakeholder-specific and cross-group themes were identified towards development of the results and discussion sections that follow.

## Results

A total of 20 participants, representing SUNCEP, education, and parent and caregiver stakeholders, participated in key informant interviews or focus groups. This section is organized by response categories and stakeholder type. Representative quotes are infused to illustrate the significance of respondent perspectives.

### Reflections on Changes since AHEC Inception

*SUNCEP Stakeholders*. The SUNCEP stakeholders reported that the type of communication for teacher/learner selection changed since AHEC integration. Communication that previously had been initiated by SUNCEP or school administrators now involved increased district participation in teacher and learner recruitment and selection. The first year’s efforts included review of national performance levels and assessment of rural versus non-rural schools and communities. Because of district “buy in”, the program model is now well recognized and growing.

*Education Stakeholders*. Tutors of seventh and eighth grade AHEC learners stated that they are able to teach and manage their classrooms differently when teaching AHEC students as compared to their regular students. A smaller class size and more advanced students were mentioned as reasons for this difference. Tutors who are part of the 10th to 12th grade program stated that they have little to no contact with parents of students in the program. Alternatively, tutors for grades 7–8 had more contact with parents.

### AHEC Greatest Progress to Date

*SUNCEP Stakeholders*. Staff reported progress in stakeholder buy-in by students, parents, and tutors related to program implementation. Staff described the progress as “new beginnings” and cited activities that supported this progress. Selected responses related to these results are included below:

“People quickly saw the benefit of the program which helps with buy-in.”

“[We are] getting almost 100% attendance at parent meetings.”

“Teachers are coming to professional learning sessions over weekends.”

*Education Stakeholders*. For tutors who had been a part of the program for more than one year, seeing their students progressing and being successful were regularly cited. Some of the tutors who were new to the program stated that having fewer “bad” behaviour issues and having students willing and ready to learn was a good accomplishment. Hearing positive feedback from those in the community was also mentioned as one of the greatest progress areas to date. Selected responses related to these results are included below:

“They [students] e-mail me and said no, the final exam was good. And I’m really proud of myself about that. I must be proud of myself because I did something right.”

“[I] see learners achieve and doing great. And kind of let me say people are talking about it – yeah … weekends I normally go fishing, and I meet people. And so we started talking and they would say – talk about the program, and I say, okay, yes, I know the program.”

*Parent/Caregivers Stakeholders*. Parents’ reported satisfaction with the AHEC program was high. Parents expressed that the most important parts of the AHEC program were the motivation that the program gave their child to succeed and the opportunity the program provided for their children. By participating in the AHEC program, they noted, their children were not limited to their parents’ financial resources or the community in which they lived. Parents observed that their children were more focused on their school work and that their grades improved as a result of participating in this program. Selected responses related to this result are included below.

“For my son, it was a huge motivation ’cause his marks increased last year in math from about 14 to 17. He’s more committed to it. He loves to study.”

“And at the end of the day, it’s an opportunity for our children, my daughter in particular, to achieve that goal that she had since age six. Like she says, she’s always told us at a young age she wants to become a doctor. And now she’s been given the platform to start reaching out to [achieve that goal].”

“It’s given her lots of self-confidence, and it’s given her motivation.”

### AHEC Challenges

*SUNCEP Stakeholders*. Program timing and logistics were frequently cited challenges. The program initially started late in the school year, which meant administrative and teaching staff were charged with developing their roles and responsibilities within a truncated time period. Logistically, participants in and from rural areas had to travel great distances in order to participate. This created a hardship for a number of learners, their parents, and their tutors. Representative responses are included below:

“Increasing the contact time at holiday schools. At the holiday schools, we see students for five consecutive days, and then won’t see them again for another two months [until the next holiday]. This is too long a time for students to go with no contact.”

“Locations are more dispersed [rural areas – up to 400 kilometres/250 miles between schools], so getting the parents together is much more difficult than in grade seven intervention, where all of the schools are located in similar areas.”

*Education Stakeholders*. Tutors and curriculum advisors discussed challenges related to their new roles with AHEC. Tutors indicated that lack of familiarity with the students in the program includes current school performance in addition to the general rapport they have through more extended daily interaction as noteworthy. The biggest challenge cited by curriculum advisors was the travel cost for students who lived far from the site of the holiday school. Additionally, advisors noted that communication from students’ regular schools was poor, making it difficult to arrange food and lodging. Selected responses related to these results are included below:

“I need the schools to really realize that say [they should tell me] the fact around the numbers – the total of learners and that they should step up their feedback to me. That is one of the greatest challenges, I think.”

“I don’t know what their marks [are at] school. I thought that that might be something that we could work from, but [it is not] … But it would be good to see them.”

*Parents/Caregiver Stakeholders*. When parents were asked to discuss things that make participating in the AHEC program difficult, conflicts with other activities were cited. Parents generally placed a high priority on the AHEC program and were forced to find alternatives to the activities with which it conflicted. Selected responses related to this result are included below.

“So, there are clashes with other activities at the school, but we as parents wanted to – this is more important to take part in this program than anything else that’s happening on Tuesdays or Thursdays at the school. Like, for instance, she had a leadership meeting, which she had to miss because of this program, which did agitate her somewhat. But we’ve dealt with that and said that this program is more important. That meeting will have to take place some other time, or we’ll have to catch up some other way.”

### Political, Educational, Historical, Economic, and Cultural Realities Critical to Working with Constituents

SUNCEP and education stakeholders also discussed the relevance of South African history, politics, education, economics, and cultural realities and challenges. The historical implications of class, race, and economic realities of the present were positioned as related to challenges in the educational system and the need for AHEC. Most learners were from low-income or working-class families, with parents who sacrificed their wants for their children’s best interests. Other issues that were positioned as both community and school problems were gangs, violence, and drugs. Most directly related to AHEC were questions related to how certain groups were apparently selected to participate in the programs and others were not. Selected responses from SUNCEP and education stakeholders related to these results are included below:

“Crisis in education in South Africa.” (SUNCEP Stakeholder)

“Big divide between the well-performing schools and the schools that are not doing that well.” (SUNCEP Stakeholder)

“Teacher professional training [is important] because a lot of teachers that are coming from those schools are sometime de-motivated.” (SUNCEP Stakeholder)

“I think through all the processes that SUNCEP is involved, part of what we’re doing is to make access to the University of Stellenbosch possible for people who were excluded in the past.” (Educational Stakeholder)

*“Communities are not affluent – low SES.” (Educational Stakeholder)*.

“For instance, if you look at the communities nowadays, we have problems with violence, gangsterism, and especially what is really killing society, especially in Cape Town and in [Malmesbury], is what they call meth. It’s really killing people, and what children are doing to get money for the drug – wow. So, on the one end, it’s great opportunities for learners to be part of this program and keep them away from, say, the violence and stuff, and keep them out to realize there is a better life after all. Because if you take a look at our school now, it’s a lot of learners – it’s as if they have lost hope, so they just go [drop out of school].” (Educational Stakeholder)

### Student Interviews

Most student results were associated with their general impressions of the program. Learners discussed and detailed perceived benefits of the program, which included improved marks, the ability to achieve goals, investment in their future success, and the opportunity to receive a bursary for university. Perceived benefits included the tutors’ styles of teaching, different methods that are taught for solving problems, the ability to build relationships with learners and tutors, the challenge the program provides, and the sheer amount of learning that takes place during the program. Recommendations towards proposed improvements in the program included wanting see more children having the opportunity to experience the program, based on their own satisfaction as participants. They also described the transport they currently have (or lacked) as being a challenge. Learners also cited the need to add more experiments and use of technology or visuals to prepare them for university.

### Outcomes

Of 165 AHEC Programme alumni, 83 (50.3%) were known to be enrolled in institutions of higher education in 2017 (78 at Stellenbosch; the others at the University of Western Cape, University of Cape Town, or Cape Peninsula University of Technology). Of the 85, 11 were pursuing an MB, ChB degree, and 8 were in other health profession bachelor degree programmes. Other BSc degrees were sought by 17 students, and engineering degrees were pursued by 16 students. The other 32 were enrolled in a wide variety of programmes.

Eleven alumni were classified as prospective students at Stellenbosch. The remaining 71 were lost to follow-up; they were not enrolled at Stellenbosch but might be enrolled in another institution of higher education.

## Discussion

Key stakeholders all noted multiple program successes. Teachers and tutors seemed to benefit from training and collaboration. Parents, tutors, and SUNCEP staff saw increased maturity, academic advancement, and increased motivation amongst students.

However, there were challenges as well. Program timing (i.e. starting late in the academic/school year) was described as difficult for stakeholders, district officials, and tutors. Limited academic time and the location of some holiday schools created teaching and other logistical challenges for parents, as well as for tutors and district officials. Continuity of curriculum development and skill measurement were areas of concern expressed across interviewees. Educators believed their efforts with learners would be impacted by the new Curriculum Assessment Policy Statements (CAPS) curriculum and related requirements associated with standardized testing. Subsequently, major strides were taken so that all tutors teach using CAPS.

Communication was also a prominent theme throughout the educator interviews. Tutors longed for more communication directly from AHEC leadership, and advisors desired better communication from the schools that they served. Tutors specifically mentioned not being informed of their students’ progress as a challenge. Tutors also complained that they received no communication from the schools about the students participating in the program; hence, tutors have no information about the students’ abilities and must spend time establishing a baseline before they can begin teaching. Moreover, soon after this evaluation, several workshops were held on a quarterly basis to ensure communication among all stakeholders in the program.

Stakeholders made recommendations that included increased program awareness; steps to address social, economic, and political challenges; program restructuring; increased communication amongst stakeholders; and opportunities for additional time with students. Cross-cutting recommendations cited by at least two stakeholders groups are included in Table 5.

**Table 5:** Stakeholder Recommendations: Themes and Selected Quotes.

Themes	SUNCEP	Educator	Parent/Caregiver

Increased Program Awareness		Yeah, just to make them [parents and learners] understand what really the program is about and [what it’s leading to]. And yeah, what opportunities there are for the learners.	Do they have to attend Stellenbosch University after they finish the program? What is the criteria to become part of this programme as far as schools are concerned? Will this program carry on, or is it just for now?
Communication		So my biggest concern is more communication with the tutors. I don’t know what their marks [are at] school. I thought that that might be something that we could work from. That would be great.	Is there at any stage where we as parents are informed of their progress – what’s happening in, say, two months’ time or three-months’ time?
Program Restructuring	Consider one-day contact on a weekend during two months between holidays. For 9th and 10th grade, mathematics scores decline. We set up a generic mathematics test. We invite 60 of the top learners to take a 3-hour mathematics test and a 2-hour science test. From that we will select our top 20 students. We feel this is a much better assessment/selection tool. Now students will be able to continue along in pipeline.	You see the pupils: they’re tired. Stretch the program so that we can have more valuable time with them instead of rushing everything down on them.	So they need more experiments in natural sciences, because at school they just do written work. To enhance them, they should do more practicals [experiments] here in natural science.
Historical/Political/Contextual Factors	[Determine] what partners support this investigation of defining underserved/disadvantaged.	More time with the students – definitely more time. We can’t do miracles in 15 days of the year, 15 days of contact sessions.	

With at least half of the alumni having achieved university admission, the program must be deemed an early educational success. From the perspective of a medical educator, it might be disappointing that more students are pursuing engineering than medicine, but South Africa also has a shortage of engineers [16]. Of course, it will be several years before it can be known whether the students successfully completed their courses of study, received their degrees, and are practicing where they are most needed.

The United States and South Africa have much in common: a shortage of physicians and other health professionals, particularly in rural and impoverished areas; population groups who have suffered from discrimination and bear a disproportionate burden of disease; and an under-representation of these same groups in the medical and health professions workforce, even though physicians who are members of these groups are more likely to serve those groups.

There are important differences as well: health indices in the United States are much better than those in South Africa; shortages of personnel are less severe; and South Africa suffers from the emigration of many of its physicians, whereas the United States is one of the beneficiaries of this “brain drain”.

Although AHEC is intended to address the problems that the two countries have in common, the models upon which the programs are built are somewhat different. The major difference is in the nature of the remote site. US AHEC centres, whether attached to regional hospitals or established as separate corporations, are independent of the affiliated medical school [17]. The rural clinical school (RCS), by contrast, is based at a mini-campus of the Stellenbosch University Faculty of Medicine and Health Sciences. US AHEC centres have an advisory board (hospital model) or a board of directors (independent corporation) that usually represent local health institutions and usually include elected officials and other consumers. Board members play an important role in identifying and mobilizing local resources, building goodwill with the community, and providing guidance to the academic faculty members who are overseeing the educational program. There is no equivalent body for the RCS, but both the AHEC project and the parent SURMEPI project had advisory boards. These boards were comprised of university faculty members, a representative of Morehouse School of Medicine, and (in the case of the AHEC Advisory Board) representatives of the Western Cape Province Departments of Education and Health.

This is not to say that one model is superior to the other. It would be inappropriate to impose the US model on the South African team, although the goal of building a pipeline is the same in both countries. It might be argued, though, that whereas the SU-AHEC program was an educational success, some of the logistical problems could have been prevented had there been a local voice or voices on the advisory board to point them out before they occurred.

### Limitations

Although the views of AHEC program participants are documented here, limitations of this study are acknowledged. Due to constraints of convenience, proximity, and time, not all AHEC sites were engaged for participation in key informant interviews and focus groups, limiting the degree to which results are representative of those AHEC sites not visited by the evaluators and do not necessarily reflect the views of all stakeholders. Additionally, in the parent groups there was a language barrier, which required the use of an interpreter. The interpreter sometimes added clarifications or additional comments, but this was not consistent across groups.

## Conclusion

The SU-AHEC demonstrated its strength in building the proximal end of the health professions pipeline. The parents (mostly coloured) who participated in the program demonstrated great motivation to prepare their children for university entry. There were logistical obstacles in the low-income rural areas where the program took place, but participants did their best to overcome them. The children will be tracked over the next few years to determine whether they are able to complete their health professions studies and return to the communities where they grew up, or to similar communities, in order to help address disparities in health, health services, and health professions. South Africa is classified as a middle-income country and thus has more resources to devote to programmes of this type, and it has fewer impediments compared to less developed countries. However, if the program is successful in South Africa, it may be possible to adapt the model appropriately and create similar pipeline programs in other sub-Saharan African countries. There are factors that distinguish the South African AHEC model from that of the United States. The strengths and limitations of the two models must be taken into consideration in developing programs in other countries.
